# PD-1, PD-L1 and cAMP immunohistochemical expressions are associated with worse oncological outcome in patients with bladder cancer

**DOI:** 10.1007/s00432-022-04262-0

**Published:** 2022-08-16

**Authors:** Giorgio Ivan Russo, Nicolò Musso, Arturo Lo Giudice, Maria Giovanna Asmundo, Marina Di Mauro, Paolo G. Bonacci, Mariacristina Massimino, Dalida Bivona, Stefania Stefani, Elisabetta Pricoco, Matteo Ferro, Massimo Camarda, Sebastiano Cimino, Giuseppe Morgia, Rosario Caltabiano, Giuseppe Broggi

**Affiliations:** 1 Urology Section, Department of Surgery, University of Catania, 95123 Catania, Italy; 2 Department of Biomedical and Biotechnological Sciences (BIOMETEC), University of Catania, 95123 Catania, Italy; 3 Department of Medical and Surgical Sciences and Advanced Technologies “G. F. Ingrassia”, Anatomic Pathology, University of Catania, 95123 Catania, Italy; 4 Department of Experimental Oncology, Mediterranean Institute of Oncology (IOM), 95029 Catania, Italy; 5 Department of Urology, European Institute of Oncology, IRCCS, Milan, Italy; 6 STLab S.R.L., Catania, Italy

**Keywords:** Immunotherapy, Bladder cancer, NGS, Genes, PD-1, Tumor-infiltrating lymphocytes

## Abstract

**Purpose:**

In this study, we aimed to identify prognostic factors of cancer mortality in patients who received radical cystectomy and to identify genomic alterations in a sub-cohort of patients with locally advanced (pT3-4) and/or positive lymph nodes bladder cancer (BC).

**Methods:**

We collected 101 BC samples from 2010 to 2018 who previously received radical cystectomy. Immunohistochemical slides were evaluated for PPAR, cAMP, IMP3, Ki67, CDK4, POU5F1, Cyclin E and MDM2, p65, CD3, CD4, CD8, CD20, CD68, CD163, FOXP3, PD-1 and PD-L1 expression. We calculated a prognostic score (PS) based on the positivity to PD-1, PD-L1 and of cAMP (final score ranging from 0 to 3). DNA of each sample have been used for sequencing by NGS in a sub-cohort of 6 patients with locally advanced (pT3-4) and/or positive lymph nodes BC.

**Results:**

PD-1 ^+^ (HR [hazard ratio] 2.59; *p* = 0.04), PD-L1^+^ (HR = 6.46; *p* < 0.01) and cAMP^+^ (HR 3.04; *p* = 0.02) were independent predictors of cancer-specific mortality (CSM). Increase of PS (score = 0 as reference) was associated with CSM, 0.81 (*p* = 0.80), 4.72 (*p* = 0.01) and 10.51 (*p* < 0.0) for PS 1, 2 and 3, respectively. ERBB2 was the gene most frequently mutated.

**Conclusion:**

BC exhibited heterogenous protein expression and variable genomic features. Identification of expression of PD-1, PD-L1 and cAMP could help in predicting oncological outcomes.

**Supplementary Information:**

The online version contains supplementary material available at 10.1007/s00432-022-04262-0.

## Introduction

Bladder cancer (BC) represents the fourth most frequent neoplasm in males and the eight cause of cancer mortality in 2021 (Siegel et al. 2021).

Nevertheless, bladder cancer appears to be a heterogeneous disease since it is characterized by the presence of various pathological prognostic factors. However, the underlying mechanisms are still being discovered and, therefore, BC characterization studies are critical.

Interestingly, Wang et al. found that expression of altered cell cycle regulators (*p53*, *Rb*, and *p21*) was associated with increased risk of cancer recurrence (Wang et al. 2021).

For this reason, nowadays, several studies aimed to develop BC characterization, through cytogenetic examination, PCR, hybridation, DNA extraction and consequent genome profiling. Among these, also immunohistochemistry (IHC) represents the most widely used evaluation technique and especially when is coupled to tissue microarray (TMA) technology, they may obtain rapid and reliable results (Broggi et al. 2021a).

The identification of different prognostic factors has in the past been used to predict the prognosis of BC, but even today some pathological mechanisms are not clear and further information is needed to implement current clinical practice (Matsushita et al. 2011).

Together with these considerations, genomic characterization of BC may help to identify alterations associated with prognosis and novel therapeutic targets and biomarkers to predict outcomes.

Ross et al. demonstrated a high frequency of clinically relevant genomic alterations among urothelial carcinomas of the bladder, with 93% of tumor specimens exhibiting at least one actionable alteration with a mean of 2.6 potentially actionable alterations per tumor. The most common potentially clinically relevant genomic alterations found include the genes, *CDKN2A* (34%), *FGFR3* (21%), *PIK3CA* (20%), and *ERBB2* (17%), a finding similar to the TCGA experience with somewhat different frequencies (Ross et al. 2016).

Based on all these premises, we aimed to identify prognostic factors in patients who received radical cystectomy and to identify genomic alterations in a random sub-cohort of patients with locally advanced (pT3-4) and/or positive lymph nodes disease BC.

## Materials and methods

We collected 101 BC samples from 2010 to 2018 who previously received radical cystectomy at the Urology section of the University of Catania. This study received consent from the participants and it was conducted in accordance with the Declaration of Helsinki. The study was approved by the Local Ethics Committee (#05/2020). From each patient, we have collected clinical and pathological features. High-risk BC was considered as the presence of locally advanced (pT3-4) and/or positive lymph nodes disease while low-risk BC as the presence of organ confined (pT1-2) and negative lymph nodes, as reported by the European Association of Urology Guidelines on Muscle-invasive and Metastatic Bladder Cancer (Witjes et al. 2021).

### Tissue microarray construction

All specimens were stained by hematoxylin and eosin to find the representative cores for the tissue microarray construction. For the TMA construction, the Galileo TMA CK3500 (Integrated System Engineering, Milan, Italy) was used as previously described. This instrument is associated with an X–Y–Z automated stage that allows one to directly place selected tissue cores in the recipient TMA block containing premade holes, ensuring not only a significant reduction in the array construction time but also an extreme alignment accuracy (Cardano et al. 2013).

### Immunohistochemistry (IHC)

Immunohistochemical slides were evaluated by two pathologists (G.B and R.C.) with no information on patient clinical data. Intensity of staining (IS) was graded on a 0–3 scale (0 = absent staining, 1 = weak staining, 2 = moderate staining, 3 = strong staining) as previously described (Broggi et al. 2021a, b).

For the Peroxisome proliferator-activated receptor (PPAR)(SC7273), cyclic adenosine monophosphate (cAMP) (ab76238), Insulin-like growth factor II messenger ribonucleic acid (mRNA) binding protein 3 (IMP3) (ab179807), Ki67 (Dako, Glostrup, Denmark), CDK4 (SC23896), POU class 5 homeobox 1 (POU5F1) (SC8629), Cyclin E (SC247) and murine double minute-2 (MDM2), p65 (SC8008), CD3 (Dako, Glostrup, Denmark), CD4 (Dako, Glostrup, Denmark), CD8 (Dako, Glostrup, Denmark), CD20 (Dako, Glostrup, Denmark), CD68 (Dako, Glostrup, Denmark), CD163 (Cell Marque Corporation- Sigma Aldrich, California, USA), forkhead box p3 (FOXP3) (SC53876), PD-1 (ab52587) and PD-L1 (ab205921) expression, the scoring system included an analysis of staining intensity (IS) as previously described (Russo et al. 2020; Broggi et al. 2020). Negative control slides were obtained by incubating them with phosphate-buffered saline (PBS) instead of the primary antibody.

We evaluated the immunopositivity for cAMP, IMP3, Ki67, CDK4 (Cyclin-dependent kinase4), POU5F1, Cyclin E, MDM2, p65, FOXP3 and PD-L1 on bladder tumor cells, while the immunoreactivity for PD-1, CD3, CD4, CD8 and CD20 has been assessed on tumor infiltrating lymphocytes. Finally, immunostainings for CD68 and CD163 have been evaluated on tumor-associated macrophages. Cells were considered as positive if brown chromogen was detected within cytoplasms/cell membranes.

### DNA analysis in a random sub-cohort of patients (*n* = 6) with locally advanced (pT3-4) and/or positive lymph nodes BC

Samples were delivered in the form of paraffinized tissue samples. For each patient, a portion of healthy tissue and a portion of tumor were removed.

The deparaffinization was carried out following the protocol provided by Qiagen with modifications. The paraffin drums containing the samples were initially treated in such a way as to eliminate any excess of pure paraffin. They were then incubated in 400 µL of Xylene for 8 min and vortexed. The suspension obtained was centrifuged for 90 s at maximum speed. The supernatant was removed, and the procedure was repeated a second time. The samples were immersed for 5 min in 400 µL of Absolute Ethanol, subjected to vertexing and centrifuged at maximum speed for 90 s, after which the supernatant was removed. This step was also repeated a second time. The last deparaffinization step involves immersion in 400 µL of Nuclease Free Water for 5 min, vertexing and centrifuging at maximum speed with subsequent removal of the supernatant. Repeated this step, the recovered tissue was stored at − 80° C.

DNA was extracted from the tissue obtained with Deparaffinization Procedure using the QIAamp DNA Mini Kit (Ref. 51304, QIAGEN, 40724 Hilden, Germany) according to the manufacturer’s instructions. DNA was quantified using the fluorimeter Qubit dsDNA BR Assay Kit (Invitrogen, Carlsbad, CA, USA; cod. 32850). The results of quantification are shown in Suppl. Table 1.

### NGS sequencing

Eighty nanograms of DNA of each sample have been used for sequencing by NGS. The NGS sequencing was carried out according to the manufacturer’s instructions provided in QIAseq Targeted DNA Panel Handbook 02/2020 in the Molecular Biology laboratory of University of Catania on a MiSeq platform (Illumina) with the Panel “QIAseq Targeted DNA Panel DHS-101Z-12”, this panel sequenced hot spot region in several (*n* = 174) of 23 genes often mutated in cancers: *NRAS, ALK, IDH1, MKRN2, RAF1, CTNNB1, FOXL2, PIK3CA,PDGFRA, KIT, ESR1, EGFR, MET, BRAF, GNAQ, RET, KRAS, ERBB3, AKT1, IDH2, TP53, ERBB2, MIR4728, GNA11*. Denature and dilute libraries were performed following the “Denature and Dilute Libraries Guide” protocol provided by Illumina^®^ (Document # 15039740 v10). Finally, sequencing was performed using the MiSeq Reagent Kits v2 (Ref. 20135740, Illumina, Inc. San Diego, CA 92122 USA).

### Statistical analysis

The mean and median of tissue protein stainings, expressed as a percentage of immunoreactive cells, will be calculated. Continuous variables will be presented as median and interquartile range (IQR) and were compared by the Student-independent *t* test or the Mann–Whitney *U* test based on their normal or not-normal distribution, respectively (normality of variables’ distribution was tested by the Kolmogorov–Smirnov test). Categorical variables will be tested with the Chi-square test.

CSM was defined as time from random assignment to death resulting from bladder cancer, and overall mortality as time from random assignment to death resulting from any cause (Kamat et al. 2016).

Kaplan–Meier curves were applied to calculate cancer-specific mortality and log-rank test was used to verify statistical significance between curves.

Cox regression analysis has been performed to test independent variables associated with cancer-specific mortality. All statistical analyses were completed using Stata software, version 14 (StataCorp. 2015. Stata Statistical Software: Release 14. College Station, TX: StataCorp LP.).

Mutational analysis was performed using QIAGEN CLC Genomics Workbench 21 software and following the User Manual for CLC Genomics Workbench 21.0.3, released on January 25 2021 (QIAGEN Aarhus, Silkeborgvej 2 Prismet DK-8000 Aarhus C, Denmark). In this step, the fast data generated by the Miseq run are processed, aligned to the reference genome hg39, filtered for quality parameters and exported as non-annotated CSV.

Further information on the mutations obtained, the amino acid changes, the impact on the protein structure and the dbSNP_IDs, was used the online tool PROVEAN®, to provide predictions for any type of protein sequence variations including the following.

Clinical considerations, population incidence (MAF) and genomic information were obtained from https://www.ncbi.nlm.nih.gov/snp/.

For all statistical comparisons, a significance level of *p* < 0.05 will be considered to show differences between the groups.

## Results

Table 1 shows the baseline characteristics of the cohort. Median age was 72.0 (IQR [interquartile range] 63.0–78.0) and median follow-up was 36 months (IQR 13.0–36.0). Forty-three (42.57%) patients had organ confined (pT1-2) disease, 54 (53.47%) had locally advanced (pT3-4) disease and 28 (27.72%) had positive lymph nodes.

**Table 1:** Baseline characteristics of the study cohort (*n* = 101)

Age, median (IQR)	72.0 (63.0–78.0)
Smoking status, *n* (%)	
No smoker	20 (19.80)
Former smoker	29 (28.71)
Current smoker	52 (51.49)
BCG immunotherapy, *n* (%)	
Yes	89 (88.12)
No	12 (11.88)
Pathological stage, *n* (%)	
pT0	9 (8.91)
pT1	10 (9.9)
pT2	28 (27.72)
pT3	39 (38.61)
pT4	15 (14.85)
Grade, *n* (%)	
Low	2 (1.98)
High	90 (89.11)
Cis, *n* (%)	
Present	6 (5.94)
Absent	95 (94.06)
Lymph node status, *n* (%)	
pN0	73 (72.28%)
pN1	28 (27.72%)

*IQR*  interquartile range; *BCG*  bacillus Calmette Guerin; *Cis * carcinoma in situ

Supplementary Table 2 shows the IHC positivity of each prognostic factor, while supplementary Fig. 1 shows the expression of markers in bladder cancer tissue.

When considering results of stainings, we found that CD3^+^ (OR [odds ratio] 3.33; < 0.01), CD68^+^ (OR: 2.73; *p* < 0.05), CD163^+^ (OR: 2.49; *p* < 0.05), CD8^+^ (OR: 2.32; *p* = 0.04), PD-L1^+^ (OR: 11.61; *p* < 0.01), CDK4^+^ (OR: 4.44; *p* < 0.05) and Ki-67^+^ (OR: 3.62; *p* < 0.01) were associated with greater risk in the presence of locally advanced (pT3-4) and/or positive lymph nodes disease (Table 2).

**Table 2:** Univariate logistic regression analysis for factors associated with locally advanced (pT3-4), positive lymph nodes disease or combination

	pT3-4	pN +	Locally advanced (pT3-4) and/or positive lymph nodes disease
	OR (95% CI)	OR (95% CI)	OR (95% CI)
CD3, + vs. −	3.15 (1.31–7.58)*	2.69 (0.91–7.93)	3.33 (1.39–7.98)**
CD4, + vs. −	1.85 (0.75–4.54)	1.06 (0.40–2.79)	2.30 (0.89–5.89)
CD8, + vs. −	2.31 (1.04–5.14)*	1.12 (0.47–2.68)	2.32 (0.89–5.89)*
CD20, + vs. −	1.53 (0.66–3.58)	2.29 (0.93–5.66)	2.16 (0.89–5.23)
CD68, + vs. −	2.54 (1.09–5.95)*	1.76 (0.66–4.68)	2.73 (1.16–6.38)*
CD163, + vs. −	2.77 (1.18–6.47)*	0.93 (0.38–2.33)	2.49 (1.07–5-77)*
Ki-67, + vs. −	3.24 (1.40–7.48)**	2.34 (0.88–6.20)	3.62 (1.56–8.39)**
P65, + vs. −	1.38 (0.62–3.07)	1.24 (0.51–2.98)	1.74 (0.77–3.92)
PD1, + vs. −	1.45 (0.61–3.46)	2.29 (0.91–5.74)	2.14 (0.86–5.32)
PD-L1, + vs. −	7.24 (2.48–21.12)**	3.56 (1.41–8.98)**	11.61 (3.22–41.83)**
PPAR-gamma, + vs. −	2.85 (1.06–7.63)*	0.77 (0.27–2.19)	2.31 (0.87–6.18)
cAMP, Loss vs. Present	2.05 (0.78–5.35)	2.34 (0.889–6.16)	2.77 (0.99–7.74)
IMP3, + vs. −	4.69 (0.53–41.70)	2.8 (0.53–14.78)	3.96 (0.44–35.22)
CDK4, + vs. −	3.82 (0.96–15.18)	3.93 (0.87–17.72)	4.44 (1.07–18.35)*
FOXP3, + vs. −	1.8 (0.31–10.30)	15.65 (1.73–140.94)	3.96 (0.44–35.22)
B-catenin, + vs. −	0.98 (0.42–2.21)	0.90 (0.35–2.30)	1.09 (0.47–2.51)

*p < 0.05

**p < 0.01

*OR*  odds ratio; *CI*  confidence interval

At the Kaplan–Meier analysis, PD-1^+^ patients (26%) respect to PD-1^−^ (66%; *p* < 0.01), PD-L1^+^ patients respect to PD-L1^−^ (71%; *p* < 0.01) and cAMP^+^ (30%) patients respect to cAMP^−^ (62%; *p* = 0.04) had worse 3-years cancer-specific survival.

When evaluating survival analysis, we found that PD-1 (HR 2.59; *p* = 0.04), PD-L1 (HR 6.46; *p* < 0.01) and cAMP^+^ (HR 3.04; *p* = 0.02) expression were independent predictors of cancer-specific mortality (receiver operating curve [ROC]: 0.77) (Table 3).

**Table 3:** Univariate and Bivariate regression analysis for factors associated with cancer-specific mortality

	HR (95% CI)	*p* value	HR (95% CI)a	*p* value
BC stage, locally advanced (pT3-4) and/or positive lymph nodes disease vs. organ confined (pT1-2)	3.41 (1.12–10.40)	0.03	–	–
pT, pT3-4 vs. pT1-2	2.26 (0.85–6.05)	0.10	–	–
pN, positive vs. negative	3.01 (1.19–7.61)	0.02	–	–
CD3, + vs. −	4.67 (1.07–20.33)	0.04	3.76 (0.85–16.63)	0.08
CD4, + vs. −	1.06 (0.38–3.00)	0.9	–	–
CD8, + vs. −	1.67 (0.65–4.31)	0.29	–	–
CD20, + vs. −	1.40 (0.55–3.63)	0.48	–	–
CD68, + vs. −	2.28 (0.75–6.97)	0.15	–	–
CD163, + vs. −	2.38 (0.78–7.25)	0.13	–	–
Ki-67, + vs. −	4.19 (1.20–14.53)	0.02	3.25 (0.91–11.63)	0.07
P65, + vs. −	1.20 (0.48–3.05)	0.70	-	-
PD1, + vs. −	2.94 (1.16–7.46)	0.02	2.59 (1.01–6.61)	0.04
PD-L1, + vs. −	7.83 (2.91–21.08)	< 0.01	6.46 (2.20–18.96)	< 0.01
PPAR-gamma, + vs. −	1.15 (0.38–3.50)	0.80	–	–
cAMP, Loss vs. Present	3.44 (1.35–8.77)	< 0.01	3.04 (1.18–7.81)	0.02
IMP3, + vs. −	3.58 (1.02–12.49)	0.04	2.69 (0.76–9.52)	0.12
CDK4, + vs. −	1.04 (0.81–1.33)	0.75	–	–
FOXP3, + vs. −	0.90 (0.12–6.74)	0.92	–	–
b-catenin, + vs. −	1.85 (0.73–4.7)	0.19	–	–

*BC *  bladder cancer; *HR*  hazard ratio; *CI*  confidence interval; locally advanced (pT3-4) and/or positive lymph nodes; *BC*   pT3-4 and/or positive lymph nodes; Organ confined = pT1-2 and negative lymph nodes

^a^Adjusted for BC stage

**Fig. 1:**
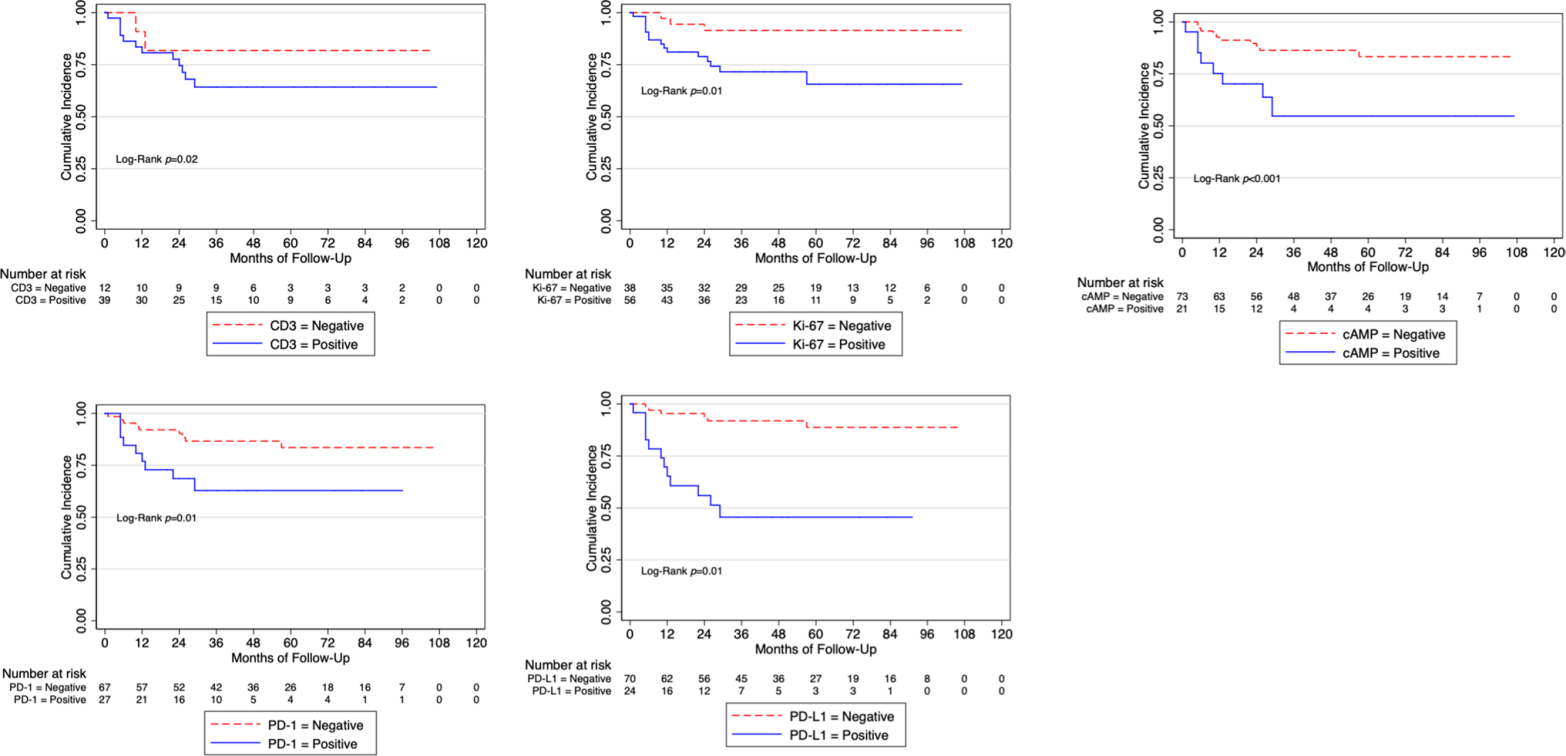
Cancer specific mortality according to CD3, Ki-67, cAMP, PD-1 and PD-L1 expression in the whole cohort. *cAMP* Cyclic adenosine monophosphate, *PD* Programmed cell death protein 1, *PD-L1* Programmed cell death protein ligand-1

**Fig. 2:**
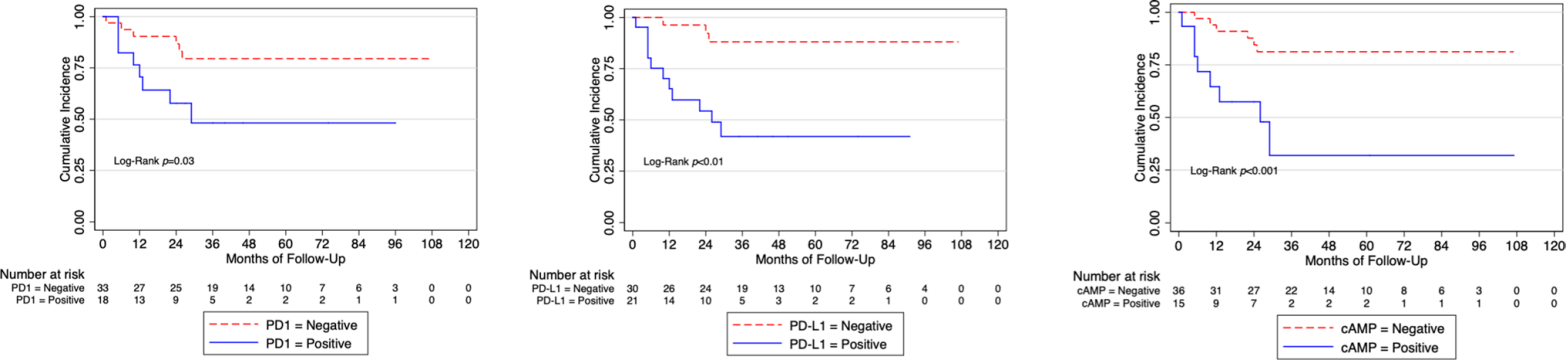
Cancer specific mortality according to PD-1, PD-L1 and cAMP expression in high-risk patients. *AMPc* Cyclic adenosine monophosphate, *PD* Programmed cell death protein 1, *PD-L1* Programmed cell death protein ligand 1

We found that patients with positive expression of PD-1, PD-L1 and of cAMP had worse cancer-specific mortality (Fig. 1) and these findings were also present in the sub-cohort of patients with locally advanced (pT3-4) and/or positive lymph nodes disease BC (Fig. 2). Supplementary Fig. 2 shows Cancer-specific mortality according to CD163, b-catenin, CD8, CD4, CD68, CD20, PPAR-gamma, FOXP3, CDK4 and IMP3 in the whole cohort.

We calculated a prognostic score (PS) based on the positivity to PD-1, PD-L1 and of cAMP, assigning 1 point based on the positivity on the immunostaining using and then summed, to calculate final score ranging from 0 to 3.

**Fig. 3:**
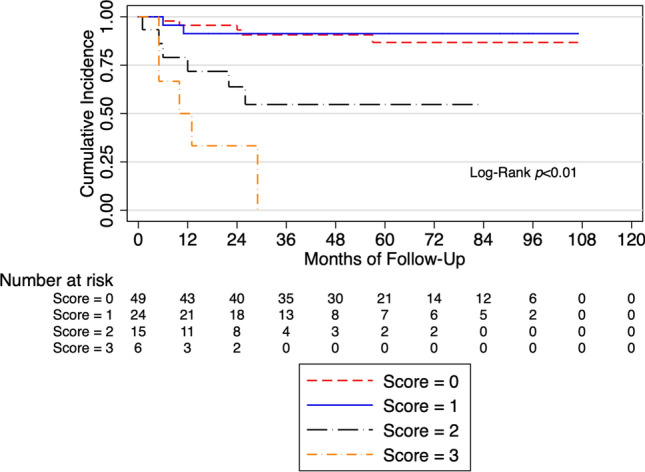
Cancer specific mortality according to prognostic score expression in locally advanced (pT3-4) and/or positive lymph nodes disease

Overall, the 36-months cancer-specific survival was 77.1% (95%CI 0.62–0.87), 51.5% (95%CI 27.98–70.76), 20.34% (95%CI 5.46–41.80) and 28.57% (95%CI 4.11–61.15 [12 months of follow-up) in patients with PS of 0, 1, 2 and 3, respectively (*p* < 0.01 at log-rank test) (Fig. 3).

The 36-months cancer-specific survival in group with organ-confined BC was 73.6% (95% CI 56.3–84.9). When stratifying for prognostic score, we reported a cancer-specific survival of 83.7% (95%CI 62.19–93.58) and of 49.7% (95% CI 16.2–76.4) (Supplementary Fig. 4). We did not observe events in score 2 and 3.

At the adjusted (for BC stage [organ confined vs. locally advanced (pT3-4) and/or positive lymph nodes disease) Cox regression analysis, we found that the increase of PS (score = 0 as reference) was associated with a significantly increase of hazard ratio (HR) of cancer-specific mortality, equal to 0.81 (95%CI 0.16–4.21; *p* = 0.80), 4.72 (95%CI 1.28–16.19; *p* = 0.01) and 10.51 (95%CI 2.66–41.44; *p *< 0.01) for PS 1, 2 and 3, respectively (ROC: 0.74).

Slides for POU5F1, Cyclin E and MDM2 have been not considered because we did not find positivity in any tumor.

### Genome sequencing

Sequencing Quality Analysis are shown in Supplementary Table 2.

All the non-synonymous mutations sequenced with the available panel that have satisfied the filters in terms of coverage and frequency are shown in Suppl. Table 3. The frequency of the sequenced mutations for which there is a reference in the literature is instead reported in Suppl. Table 4.

The sequencing in several regions of the 23 genes of interest showed 8 of them often mutated in both cancer and mucosa. These mutations were found in the following genes: *KRAS, KIT, ESR1, EGFR, ALK, PIK3CA*, *TP53* and *ERBB2*, from the lower to the higher number of mutations reported in each gene, respectively. The analysis output exhibited *ERBB2* gene as the most mutated, in particular P1170A, c.3508C > G, ***rs1058808,*** was presented in every sample except for patient 2 which showed a departed group of mutations.

Of all found SNP mutations in this sub-cohort, 36 have a known dbSNP_ID, which is a unique identifier assigned to a single nucleotide polymorphism (SNP) when it is submitted to the SNP database. The most frequent mutations are those identified with the dbSNP_ID rs1042522 and rs1058808, with a frequency of 83.3% (5 out of 6 patients). Mutations rs2227983, rs7766585 and rs1042522 were found in 50% of patients. The rs61764370 mutation has a frequency of 33.3% while all remaining mutations have a frequency of 16.7%, found in individual patients (Suppl. Table 5).

## Discussion

Herein, we reported that the expression of the PD-1, PD-L1 and cAMP was associated with a worse prognosis and in particular with a greater mortality in patients with locally advanced (pT3-4) and/or positive lymph nodes disease. In fact, although radical surgery is performed with curative intent, more than 50% of patients with pathological evidence of cancer invading through the muscularis propria or involving the regional lymph nodes will have lethal metastatic recurrence (Witjes et al. 2021).

These results, although preliminary, may offer new future scientific insights with the aim of highlighting possible predictors of prognosis in particular patients with worse adverse pathology. These findings, in fact, may serve for several aspects including the use of these markers in the context of response to chemotherapy or even investigating the role of intravesical immunotherapy in patients at high risk of progression.

Many recent evidences, together with several clinical trials, have highlighted the importance of immune system receptors for the prognosis and progression of bladder cancer.

The molecular basis for the intrinsic control of immune effector cells is based on groups of activatory (for example, CD28 and CD137) and inhibitory (for example, PD-1, cytotoxic T lymphocyte antigen 4 (CTLA-4) and B and T lymphocyte attenuator (BTLA; also known as CD272)) receptors on the cell surface (Schneider et al. 2019).

Moreover, the impact of tumor infiltrating lymphocytes (TILs) on survival was confirmed in various cancer types but the some controversies are still present (Huang et al. 2018). The evidences suggest that TILs are the representation of the relationship between our immune system and tumor microenvironment and it includes macrophages, neutrophil granulocytes, dendritic cells, mast cells, natural killer cells, naive and memory B lymphocytes and effector T cells (T helper cells; regulatory T cells; and cytotoxic T cells) (Fridman et al. 2012). Huang et al. in fact reported that patients with intense TILs were independently associated with better OS (Huang et al. 2018).

In contrast, we evaluated the expression of CD3 + and CD8 + TIL subpopulations together with FOXP3 for regulatory T cells and CD68 for macrophages and we reported that CD3 + patients were associated with worse outcomes as reported in Fig. 1.

In a study from Wang et al., that included a cohort of patients with non-muscle invasive bladder cancer (NMIBC) or muscle invasive bladder cancer (MIBC), authors demonstrated that high level of PD-L1 expression on tumour-infiltrating immune cells was an independent predictor of worse survival (Wang et al. 2019). Importantly, high PD-1 or PD-L1 expression levels in whole tumour samples might reflect strong immune cell infiltration and activation in the tumour microenvironment, which might be linked to improved patient outcomes (Schneider et al. 2019).

Due to the poor prognosis of patients with advanced BC and in consideration that, no standard adjuvant systemic therapies have been shown to improve outcomes, more and more evidences are suggesting a putative role of adjuvant immunotherapy in this category of patients. Two recent phase 3 randomized controlled trials (RCTs)—Checkmate-274 (Bajorin et al. 2021) and IMvigor010 (Bellmunt et al. 2021)—evaluated adjuvant immune checkpoint inhibitor therapy for patients with resected high-risk MIBC.

In the intent-to-treat population of CheckMate 274, disease-free survival was 21 months in patients who received nivolumab vs about half that—10.9 months—for those assigned to placebo, a 30% reduction in recurrence or death favoring nivolumab (*p* < 0.001).

In the PD-L1–positive (≥ 1%) population, the disease-free survival benefit with nivolumab was even more robust, with median disease-free survival not reached for the nivolumab-treated patients compared with 10.8 months with placebo, representing a 47% reduction in risk of recurrence or death favoring nivolumab (*p* < 0.001) (Bajorin et al. 2021).

On the contrary, IMvigor010 did not meet its primary endpoint of improved disease-free survival in the atezolizumab group over observation (Bellmunt et al. 2021). However, in the exploratory analysis, patients who were positive for ctDNA had improved disease-free survival and overall survival in the atezolizumab arm versus the observation arm (disease-free survival hazard ratio = 0.58; *p* = 0.0024, overall survival hazard ratio = 0.59). No difference in disease-free survival or overall survival between treatment arms was noted for patients who were negative for ctDNA (Powles et al. 2021).

Importantly, patients those with positive ctDNA showed correlation with high tumour mutational burden and PD-L1 from the primary tumor. Thus, the biology of tumor is strongly important to predict outcomes and response to therapy.

Our findings on cyclic AMP (cAMP) are justified by previous researches that demonstrated that cyclic nucleotides regulate a myriad of biological processes such as cell growth and adhesion, energy homeostasis, neuronal signaling, and muscle relaxation and also cancer growth (Zhang et al. 2020).

However, it is important to underline that histological heterogeneity of BC is associated with different response to systemic therapies. A typical example of the need for comprehensive biomarker assessments is represented by the role of FGFR3 alterations, which do not correlate with response to preoperative pembrolizumab in muscle invasive bladder cancer (Necchi et al. 2020). Interestingly, Necchi et al. have previously reported that FGFR3 alterations are enriched in FGFR3-active tumors based on RNA expression signature, which in turn tend to have lower immune activity, and lower PD-L1 and PD-L2 expression, suggesting that FGFR3 activity may provide a potential tool for further discriminating the mechanisms underlying the response and resistance to neoadjuvant checkpoint inhibition (Necchi et al. 2019).

Data on FGFR3 are, however, conflicting. Isharwal et al. sequenced 454 BC patients using next generation sequencing assay (MSK − IMPACT). Patients whose primary tumors harbored TP53, RB1, TP53/MDM2, and cell cycle pathway alterations more frequently presented with advanced disease whereas those with tumors containing FGFR3, and RTK/RAS/RAF pathway alterations generally presented with NMIBC (Isharwal et al. 2018).

Importantly, Yang et al. reported that receptor tyrosine kinases (ERBB2, FGFR3, and PIK3CA) were more commonly altered in the responders (*p* < 0.01) compared to the non-responders to neoadjuvant chemotherapy (Yang et al. 2018). Although we did not perform receptor tyrosine kinases in IHC, we found high frequency of ERBB2 mutations in our sub-cohort of patients with locally advanced (pT3-4) and/or positive lymph nodes disease.

Despite the heterogenicity of the mutational pattern of bladder cancer, it was possible to identify the particular genes involved: *HER2, TP53, PIK3CA, HRAS* as well as *EGFR*.

Receptors tyrosine kinase (RTK) expressed by non-neoplastic cells may also become an attractive therapeutic target, which is considered as an emerging class of innate immune checkpoints (Akalu et al. 2017). In particular, immune microenvironment is also modulated by RTK and interestingly its inhibitors are less effective in patients with a tumor highly infiltrated by CD8+ lymphocytes and associated with a high level of programmed death-ligand 1 (PD-L1) (Matsumoto et al. 2019). These findings strengthen the importance of an interaction between RTK and immune checkpoints (Pottier et al. 2020).

Furthermore, there are preliminary hypothesis that that anti-PD-1 antibody given by intravesical route will effectively treat bladder tumors while having a very good safety profile with limited systemic effects (Kirschner et al. 2021). In particular, Anti-PD-1 increases CD8 cell infiltration in tumors, particularly when administered intravesically.

Before concluding, we would underline some limitations. First, we were not be able to verify if our findings were related to BCG response before radical cystectomy. Second, a subset analysis in patients receiving immunotherapy during the follow-up was not feasible due the low number of events. Third, we did not perform RNA analysis investigating the cross link between genomic alterations and tissue protein expression.

Fourth, although almost of our patients did not receive neoadjuvant chemotherapy, we were not able to verify its impact on assessed biomarkers and long-term survival. We also limited the NGS analysis in few patients and not in the whole cohort, but further and more complex analysis will be performed in the same study cohort. Finally, as concerning the role of TILs and its differentiation between stromal and intra-tumoral, the TMA slides are not properly correct for this evaluation since they do not represent in some cases the area in which TIL are most represented rather the full slides that are more appropriate.

On the other hand, we reported a comprehensive analysis in BC patients that demonstrated that PD^+^, PD-L1^+^ and cAMP^+^ were associated with worse prognosis and that the most frequent mutations were those identified with the dbSNP_ID rs1042522 and rs1058808, with a frequency of 83.3%.

## Conclusions

Through our immunohistochemical studies carried out on bladder cancer samples, we have shown that the expression of PD-1, PD-L1 and cAMP is able to predict mortality at 3 years in patients with locally advanced (pT3-4) and/or positive lymph nodes disease. Genetic analyses carried out in NGS also revealed how somatic mutations can involve locus of different genes, and in particular the *ERBB2* alteration proved to be the most prevalent. We think that these findings give deep knowledges in urothelial cancer strengthening the importance of the use of blood-based biomarker or tissue biomarker to select patients at high risk of relapse since they are linked with cancer biology.

## Supplementary Information

Below is the link to the electronic supplementary material.

Supplementary file1 (TIFF 14826 KB)

Supplementary file2 (TIFF 14826 KB)

Supplementary file3 (TIFF 14826 KB)

Supplementary file4 (TIFF 14826 KB)

Supplementary file5 (TIFF 14826 KB)

Supplementary file6 (DOCX 15 KB)

Supplementary file7 (DOCX 14 KB)

Supplementary file8 (DOCX 20 KB)

Supplementary file9 (DOCX 23 KB)

Supplementary file10 (DOCX 16 KB)

## Data Availability

Data can be available upon reasonable request to the corresponding author.

## References

[CR1] Akalu YT, Rothlin CV, Ghosh S (2017) TAM receptor tyrosine kinases as emerging targets of innate immune checkpoint blockade for cancer therapy. Immunol Rev 276:165–177. 10.1111/imr.12522 28258690 10.1111/imr.12522 PMC5381815

[CR2] Bajorin DF, Witjes JA, Gschwend JE et al (2021) Adjuvant nivolumab versus placebo in muscle-invasive urothelial carcinoma. N Engl J Med 384:2102–2114. 10.1056/NEJMoa2034442 34077643 10.1056/NEJMoa2034442 PMC8215888

[CR3] Bellmunt J, Hussain M, Gschwend JE et al (2021) Adjuvant atezolizumab versus observation in muscle-invasive urothelial carcinoma (IMvigor010): a multicentre, open-label, randomised, phase 3 trial. Lancet Oncol 22:525–537. 10.1016/S1470-2045(21)00004-8 33721560 10.1016/S1470-2045(21)00004-8 PMC8495594

[CR4] Broggi G, Filetti V, Ieni A et al (2020) MacroH2A1 immunoexpression in breast cancer. Front Oncol. 10.3389/fonc.2020.01519 32974186 10.3389/fonc.2020.01519 PMC7471871

[CR5] Broggi G, Lo Giudice A, Di Mauro M et al (2021a) SRSF-1 and microvessel density immunohistochemical analysis by semi-automated tissue microarray in prostate cancer patients with diabetes (DIAMOND study). Prostate 81:882–892. 10.1002/pros.24185 34196424 10.1002/pros.24185 PMC8362056

[CR6] Broggi G, Salvatorelli L, Barbagallo D et al (2021b) Diagnostic utility of the immunohistochemical expression of serine and arginine rich splicing factor 1 (SRSF1) in the differential diagnosis of adult gliomas. Cancers (basel) 13:2086. 10.3390/cancers13092086 33925821 10.3390/cancers13092086 PMC8123436

[CR7] Cardano M, Diaferia GR, Falavigna M et al (2013) Cell and tissue microarray technologies for protein and nucleic acid expression profiling. J Histochem Cytochem 61:116–124. 10.1369/0022155412470455 23172795 10.1369/0022155412470455 PMC3636688

[CR8] Fridman WH, Pagès F, Sautès-Fridman C, Galon J (2012) The immune contexture in human tumours: impact on clinical outcome. Nat Rev Cancer 12:298–306. 10.1038/nrc3245 22419253 10.1038/nrc3245

[CR9] Huang H-S, Su HY-L, Li P-H et al (2018) Prognostic impact of tumor infiltrating lymphocytes on patients with metastatic urothelial carcinoma receiving platinum based chemotherapy. Sci Rep 8:7485. 10.1038/s41598-018-25944-1 29748589 10.1038/s41598-018-25944-1 PMC5945658

[CR10] Isharwal S, Audenet F, Drill EN et al (2018) Next generation sequencing of urothelial bladder cancer: Memorial Sloan Kettering Cancer Center experience in 454 patients. J Clin Oncol 36:469–469. 10.1200/JCO.2018.36.6_suppl.469

[CR11] Kamat AM, Sylvester RJ, Böhle A et al (2016) Definitions, end points, and clinical trial designs for non–muscle-invasive bladder cancer: recommendations from the International Bladder Cancer Group. J Clin Oncol 34:1935–1944. 10.1200/JCO.2015.64.4070 26811532 10.1200/JCO.2015.64.4070 PMC5321095

[CR12] Kirschner AN, Wang J, Rajkumar-Calkins A et al (2021) Intravesical Anti-PD-1 immune checkpoint inhibition treats urothelial bladder cancer in a mouse model. J Urol 205:1336–1343. 10.1097/JU.0000000000001576 33356477 10.1097/JU.0000000000001576 PMC8112465

[CR13] Matsumoto Y, Sawa K, Fukui M et al (2019) Impact of tumor microenvironment on the efficacy of epidermal growth factor receptor-tyrosine kinase inhibitors in patients with EGFR -mutant non-small cell lung cancer. Cancer Sci 110:3244–3254. 10.1111/cas.14156 31368625 10.1111/cas.14156 PMC6778652

[CR14] Matsushita K, Cha EK, Matsumoto K et al (2011) Immunohistochemical biomarkers for bladder cancer prognosis. Int J Urol No-No. 10.1111/j.1442-2042.2011.02809.x 10.1111/j.1442-2042.2011.02809.x 21771101

[CR15] Necchi A, Madison R, Chung J et al (2019) Multiple-cohort analysis investigating FGFR3 alteration as a predictor of non-response to neoadjuvant pembrolizumab (pembro) in muscle-invasive bladder cancer (MIBC). Ann Oncol 30:v358

[CR16] Necchi A, Raggi D, Gallina A et al (2020) Updated results of PURE-01 with preliminary activity of neoadjuvant pembrolizumab in patients with muscle-invasive bladder carcinoma with variant histologies. Eur Urol 77:439–446. 10.1016/j.eururo.2019.10.026 31708296 10.1016/j.eururo.2019.10.026

[CR17] Pottier C, Fresnais M, Gilon M et al (2020) Tyrosine kinase inhibitors in cancer: breakthrough and challenges of targeted therapy. Cancers (Basel) 12:731. 10.3390/cancers12030731 32244867 10.3390/cancers12030731 PMC7140093

[CR18] Powles T, Assaf ZJ, Davarpanah N et al (2021) ctDNA guiding adjuvant immunotherapy in urothelial carcinoma. Nature 595:432–437. 10.1038/s41586-021-03642-9 34135506 10.1038/s41586-021-03642-9

[CR19] Ross JS, Wang K, Khaira D et al (2016) Comprehensive genomic profiling of 295 cases of clinically advanced urothelial carcinoma of the urinary bladder reveals a high frequency of clinically relevant genomic alterations. Cancer 122:702–711. 10.1002/cncr.29826 26651075 10.1002/cncr.29826

[CR20] Russo D, Di Crescenzo RM, Broggi G et al (2020) Expression of P16INK4a in uveal melanoma: new perspectives. Front Oncol. 10.3389/fonc.2020.562074 33154942 10.3389/fonc.2020.562074 PMC7590828

[CR21] Schneider AK, Chevalier MF, Derré L (2019) The multifaceted immune regulation of bladder cancer. Nat Rev Urol 16:613–630. 10.1038/s41585-019-0226-y 31501534 10.1038/s41585-019-0226-y

[CR22] Siegel RL, Miller KD, Fuchs HE, Jemal A (2021) Cancer statistics, 2021. CA Cancer J Clin 71:7–33. 10.3322/caac.21654 33433946 10.3322/caac.21654

[CR23] Wang B, Pan W, Yang M et al (2019) Programmed death ligand-1 is associated with tumor infiltrating lymphocytes and poorer survival in urothelial cell carcinoma of the bladder. Cancer Sci 110:489–498. 10.1111/cas.13887 30548363 10.1111/cas.13887 PMC6361576

[CR24] Wang G, Black PC, Goebell PJ et al (2021) Prognostic markers in pT3 bladder cancer: a study from the international bladder cancer tissue microarray project. Urol Oncol Semin Orig Investig 39:301.e17-301.e28. 10.1016/j.urolonc.2021.01.021 10.1016/j.urolonc.2021.01.021 PMC8501538 33563539

[CR25] Witjes JA, Bruins HM, Cathomas R et al (2021) European association of urology guidelines on muscle-invasive and metastatic bladder cancer: summary of the 2020 guidelines. Eur Urol 79:82–104. 10.1016/j.eururo.2020.03.055 32360052 10.1016/j.eururo.2020.03.055

[CR26] Yang Z, Zhang R, Ge Y et al (2018) Somatic FGFR3 mutations distinguish a subgroup of muscle-invasive bladder cancers with response to neoadjuvant chemotherapy. EBioMedicine 35:198–203. 10.1016/j.ebiom.2018.06.011 29941343 10.1016/j.ebiom.2018.06.011 PMC6154770

[CR27] Zhang H, Kong Q, Wang J et al (2020) Complex roles of cAMP–PKA–CREB signaling in cancer. Exp Hematol Oncol 9:32. 10.1186/s40164-020-00191-1 33292604 10.1186/s40164-020-00191-1 PMC7684908

