# Supplementary figures and images for: PD-1, PD-L1 and cAMP immunohistochemical expressions are associated with worse oncological outcome in patients with bladder cancer
Source: J Cancer Res Clin Oncol. 2022 Aug 16;149(7):3681–90. doi: 10.1007/s00432-022-04262-0 (PMC10314864; doi:10.1007/s00432-022-04262-0)

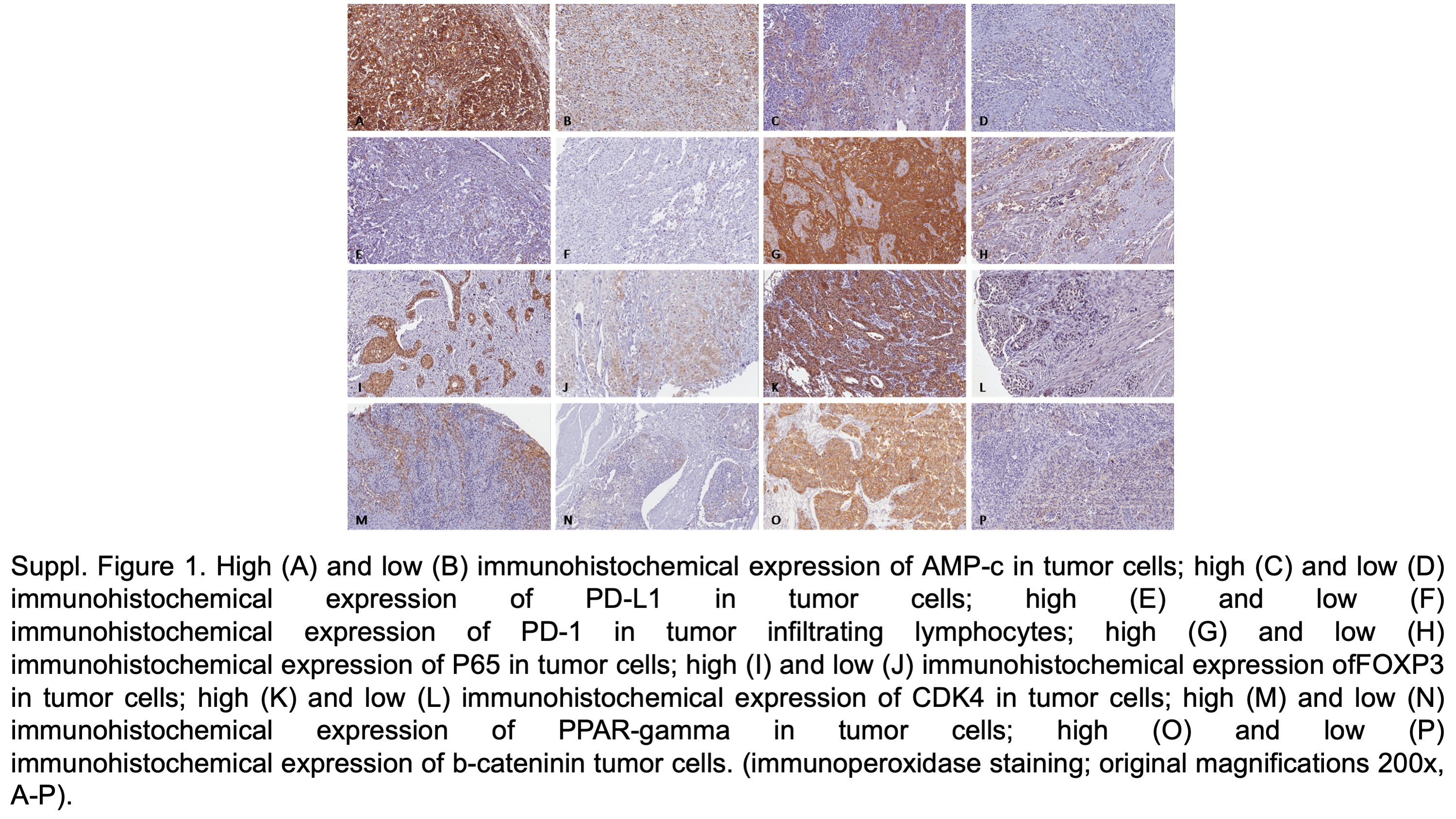

Supplement: Supplementary file 1 — Supplementary file1 (TIFF 14826 KB) [file 432_2022_4262_MOESM1_ESM.tiff]

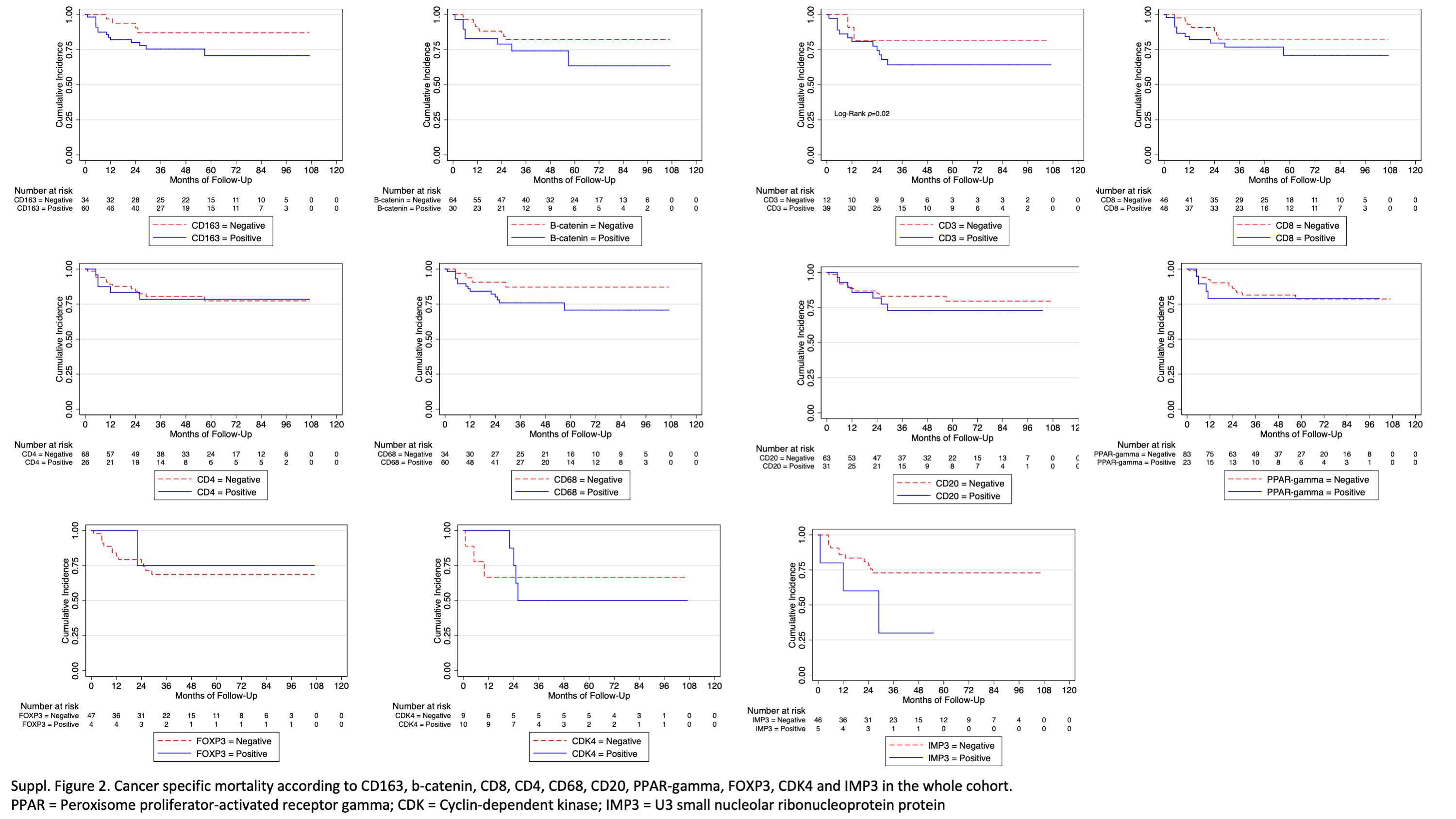

Supplement: Supplementary file 2 — Supplementary file2 (TIFF 14826 KB) [file 432_2022_4262_MOESM2_ESM.tiff]

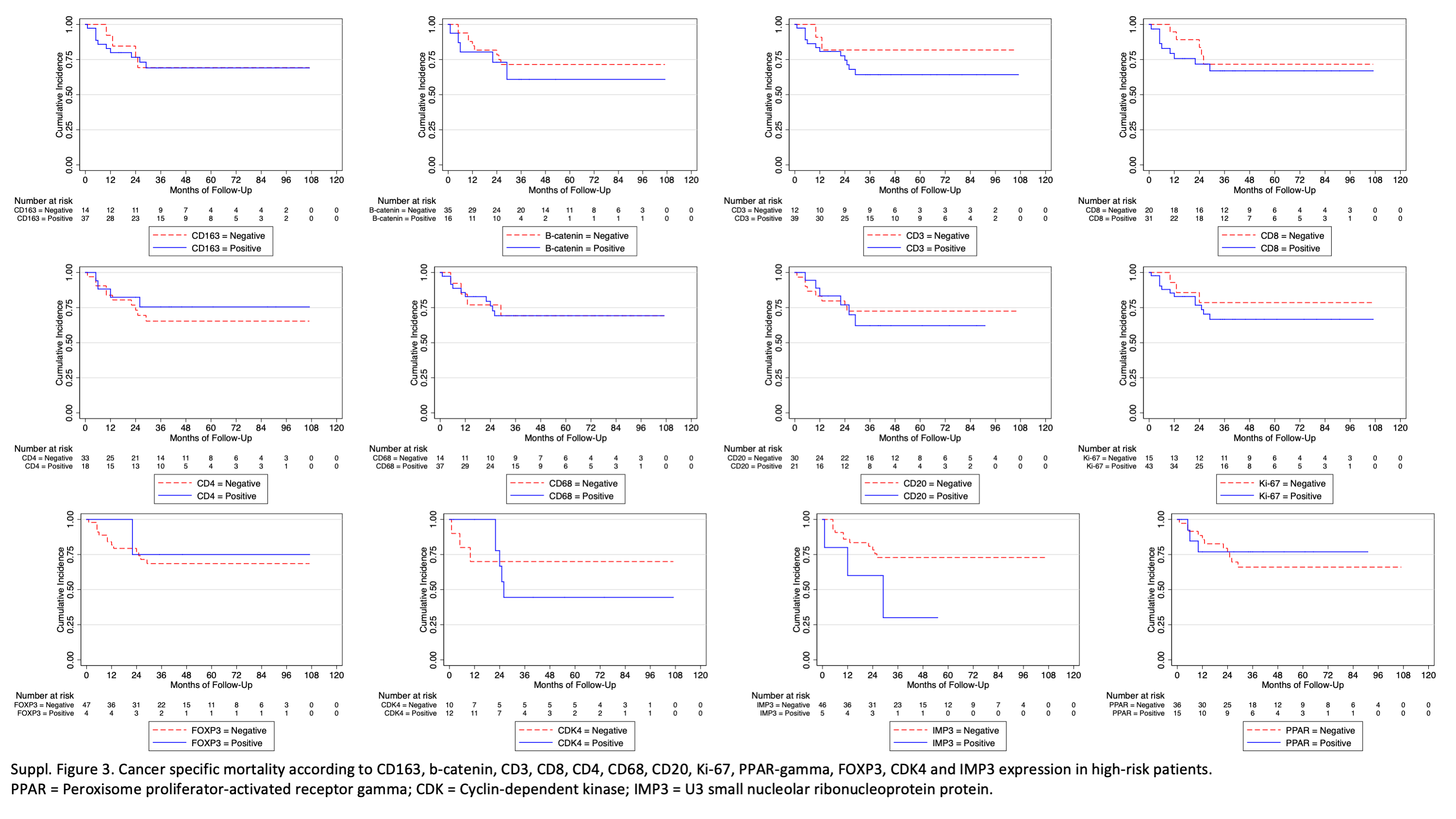

Supplement: Supplementary file 3 — Supplementary file3 (TIFF 14826 KB) [file 432_2022_4262_MOESM3_ESM.tiff]

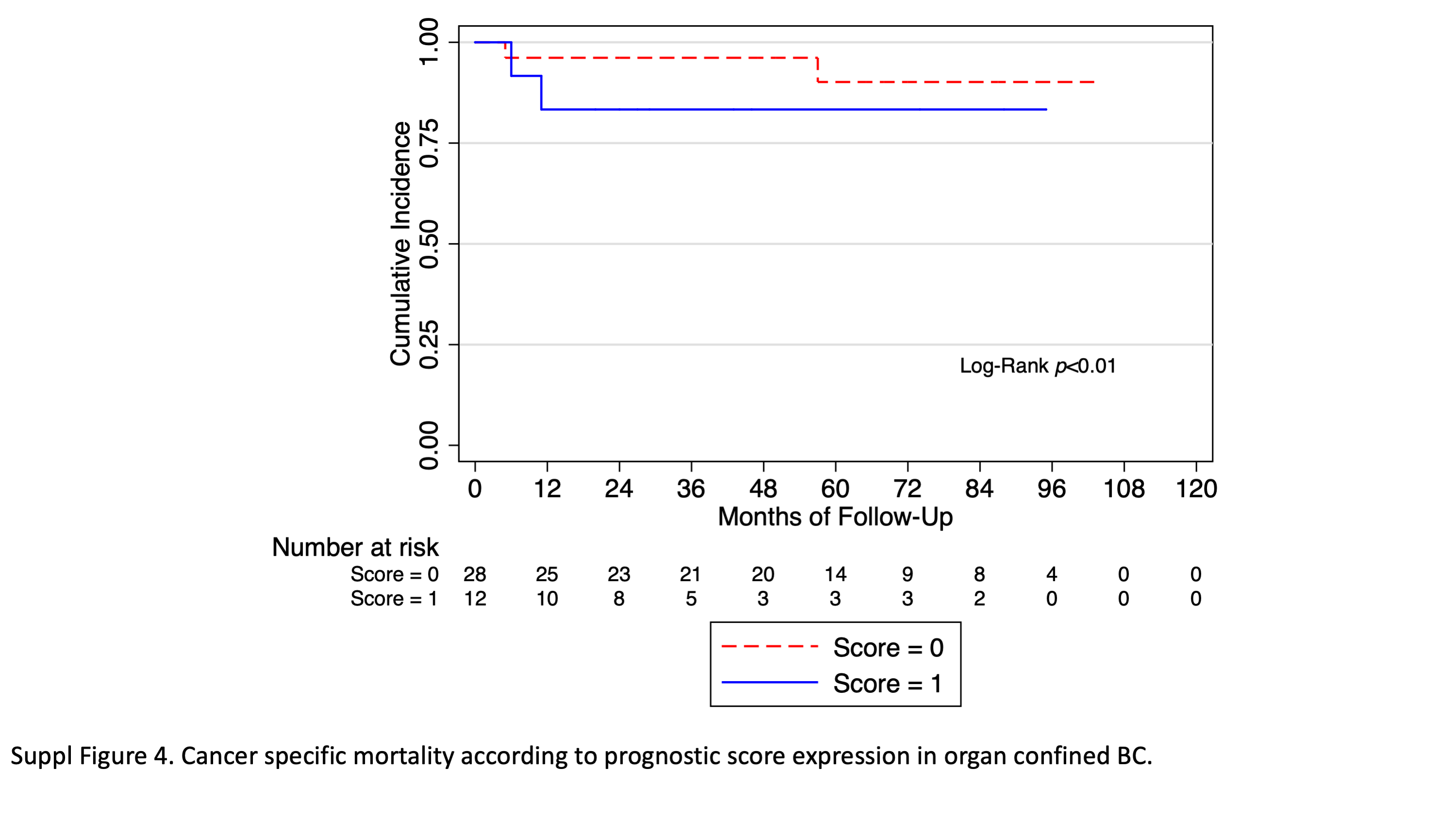

Supplement: Supplementary file 4 — Supplementary file4 (TIFF 14826 KB) [file 432_2022_4262_MOESM4_ESM.tiff]

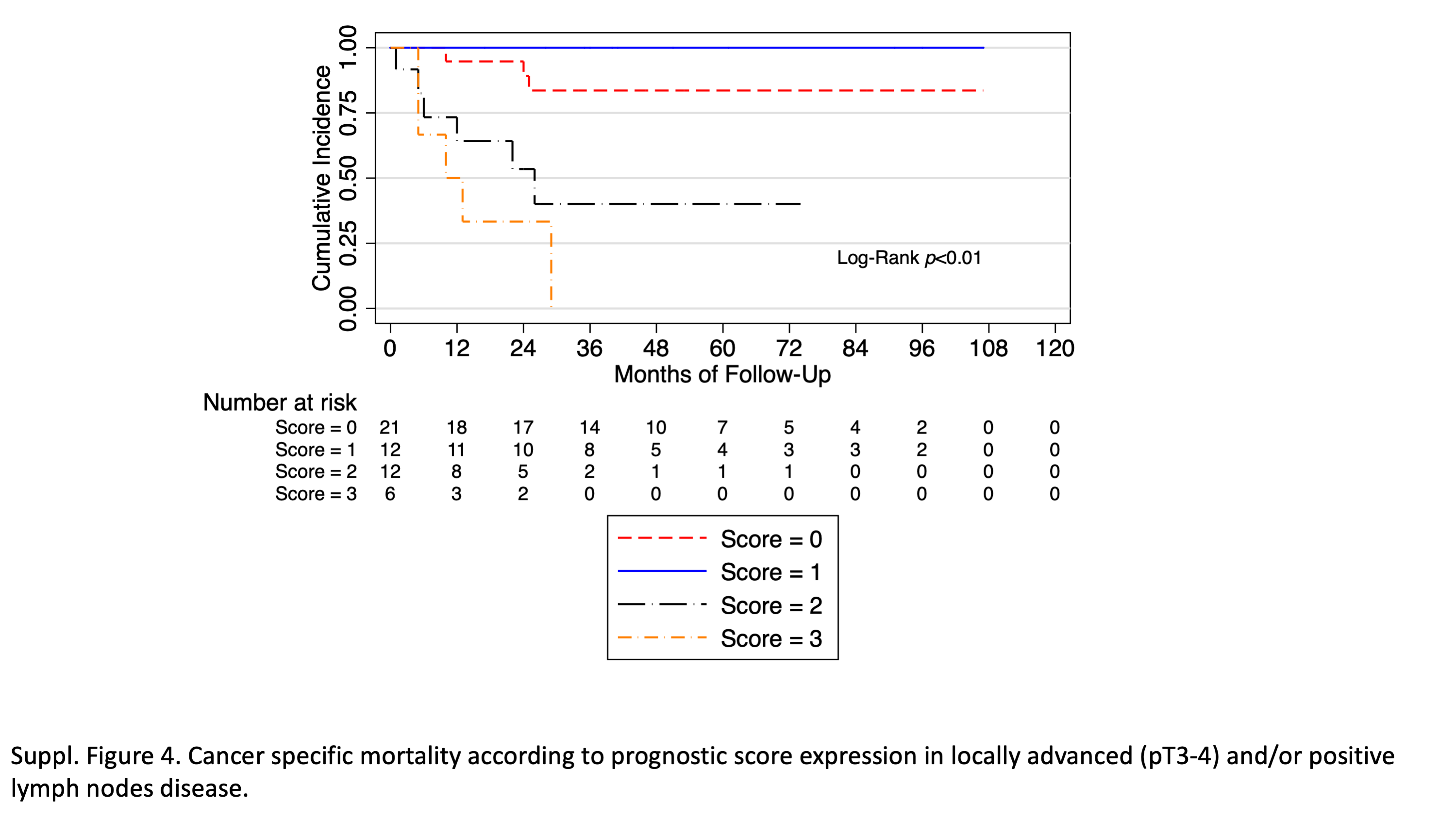

Supplement: Supplementary file 5 — Supplementary file5 (TIFF 14826 KB) [file 432_2022_4262_MOESM5_ESM.tiff]
