# Supplementary material for: PD-1, PD-L1 and cAMP immunohistochemical expressions are associated with worse oncological outcome in patients with bladder cancer
Source: J Cancer Res Clin Oncol. 2022 Aug 16;149(7):3681–90. doi: 10.1007/s00432-022-04262-0 (PMC10314864; doi:10.1007/s00432-022-04262-0)
Supplement: Supplementary file 6 — Supplementary file6 (DOCX 15 KB) [file 432_2022_4262_MOESM6_ESM.docx]

Suppl. Table 1. Samples and relative DNA dosage after extraction procedure.

|  | ***Sample*** | ***Sample Type*** | ***DNA Dosage (ng/µL)*** | ***Total DNA extracted (ng)*** |
| --- | --- | --- | --- | --- |
| ***Patient 1*** | *1-GR* | Normal | 6,52 | 293,4 |
|  | *7-GR* | Tumor | 7,96 | 278,6 |
| ***Patient 2*** | *2-GR* | Normal | 17,64 | 793,8 |
|  | *8-GR* | Tumor | 8,96 | 313,6 |
| ***Patient 3*** | *4-GR* | Normal | 7,08 | 283,2 |
|  | *10-GR* | Tumor | 3,336 | 116,76 |
| ***Patient 4*** | *5-GR* | Normal | 7,6 | 304 |
|  | *11-GR* | Tumor | 5,68 | 198,8 |
| ***Patient 5*** | *13-GR* | Normal | 8,8 | 396 |
|  | *15-GR* | Tumor | 11,48 | 516,6 |
| ***Patient 6*** | *14-GR* | Normal | 3,83 | 172,44 |
|  | *16-GR* | Tumor | 5,6 | 252 |
