# Supplementary material for: PD-1, PD-L1 and cAMP immunohistochemical expressions are associated with worse oncological outcome in patients with bladder cancer
Source: J Cancer Res Clin Oncol. 2022 Aug 16;149(7):3681–90. doi: 10.1007/s00432-022-04262-0 (PMC10314864; doi:10.1007/s00432-022-04262-0)
Supplement: Supplementary file 7 — Supplementary file7 (DOCX 14 KB) [file 432_2022_4262_MOESM7_ESM.docx]

| Suppl. Table 2. Positive expression of investigated proteins by IHC in bladder cancer. | |
| --- | --- |
| CD3 | 69 (68.32) |
| CD4 | 28 (27.72) |
| CD8 | 52 (51.49) |
| CD20 | 33 (32.67) |
| CD68 | 67 (66.34) |
| CD163 | 66 (65.35) |
| Ki-67 | 62 (61.39) |
| P65 | 43 (42.57) |
| PD1 | 30 (29.70) |
| PD-L1 | 30 (29.70) |
| PPAR | 25 (24.75) |
| IMP3 | 6 (5.94) |
| AMPc | 24 (23.76) |
| FOXP3 | 6 (5.94) |
