# Supplementary material for: PD-1, PD-L1 and cAMP immunohistochemical expressions are associated with worse oncological outcome in patients with bladder cancer
Source: J Cancer Res Clin Oncol. 2022 Aug 16;149(7):3681–90. doi: 10.1007/s00432-022-04262-0 (PMC10314864; doi:10.1007/s00432-022-04262-0)
Supplement: Supplementary file 8 — Supplementary file8 (DOCX 20 KB) [file 432_2022_4262_MOESM8_ESM.docx]

| Supplementary Table 3. Quality parameters calculated for each patient on the arithmetic mean of the mutations considered "significant" (Coverage> 150 and Frequency> 5%). | | | | | | |
| --- | --- | --- | --- | --- | --- | --- |
| **Patient** | **Sample** | **Coverage*** | **F/R Balance** | **Average Quality** | **Baseq Ranksum** | **Proportion (Singleton UMIs)** |
|  |  |  |  |  |  |  |
| **Patient 1** | **Mucosa** | 258.02 | 0.46 | 48.07 | 0.35 | 0.41 |
|  | **Tumor** | 1023.5 | 0.48 | 49.73 | 1.08 | 0.37 |
| **Patient 2** | **Mucosa** | 3346.05 | 0.47 | 45.9 | -0.95 | 0.57 |
|  | **Tumor** | 3028.5 | 0.47 | 47.63 | -0.66 | 0.51 |
| **Patient 3** | **Mucosa** | 251.36 | 0.47 | 49.44 | 0.82 | 0.36 |
|  | **Tumor** | 256.5 | 0.37 | 41.85 | 2.65 | 0.66 |
| **Patient 4** | **Mucosa** | 759.41 | 0.47 | 50.89 | 0.13 | 0.34 |
|  | **Tumor** | 114 | 0.5 | 42.5 | 1.36 | 0.66 |
| **Patient 5** | **Mucosa** | 349.74 | 0.44 | 49.11 | -0.32 | 0.39 |
|  | **Tumor** | 497.27 | 0.46 | 52.86 | -0.15 | 0.25 |
| **Patient 6** | **Mucosa** | 194.31 | 0.34 | 41.5 | 0.11 | 0.65 |
|  | **Tumor** | 314.5 | 0.46 | 48.77 | 2.08 | 0.38 |

To establish the coverage value. the number of reads can be set at the beginning. In diagnostics. the minimum acceptable level of coverage is 50x. although the yield often depends on the method and the kits used. Not infrequently it comes to 80x or 100x. Acquiring all these reads is necessary to reach a sufficient amount of signal to be picked up and cover the signal of reads that contain errors. For these reasons. the coverage filter settled for the present data was fixed at 150x. Due to a lack of data. only in patient n. 4 the coverage filter was set at 100x for the tumor. obtaining a coverage of 114. The data show very high coverage values ​​such as 3346.05 and 3028.5 respectively in the mucosa and in the tumor of patient n. 2; regarding the lowest value. it results in the mucosa of patient n. 6. with a coverage of 194.31. The higher are the values obtained. more significantly valid and supported by sequencing the data are. The F/R balance values show an equal distribution of forward and reverse reads: as foreseen by CLC protocol. values are around 0.5 except in tumor of patient n. 3. which is 0.37 and 0.34 in mucosa of patient n. 6. In the present work the Average Quality was about 40: as concern to Table 3b. our Variant Call Accuracy is between 99.9999% and 99.9%. indicating how low is the probability to produce an error. For Baseq Ranksum values. we obtain four negative ones. as -0.95 in the mucosa and -0.66 in tumor of patient n. 2. that indicate a variant with lower quality than the reference variant: meanwhile scores of 2.65 in patient n. 3 and 2.08 in patient n. 6. both in tumor. represent the highest positive values with the better quality. All Proportion (Singleton UMIs) values reveal uniformity. in a range of 0.25 and 0.66 showed in tumor. respectively in patient n. 5 and both patient n. 3 and 4.

| Supplementary Table 3b. Quality parameters table re-adapted from the CLC Genomics Workbench User Manual. |
| --- |

| **Average Quality** | **QUAL** | **Probability of incorrect  Variant Call** | **Variant Call Accuracy** |
| --- | --- | --- | --- |
| 10 | 10 | 1 in 10 | 90% |
| 20 | 20 | 1 in 100 | 99% |
| 30 | 30 | 1 in 1.000 | 99.9% |
| 60 | 60 | 1 in 1.000.000 | 99.9999% |
| - | 100 | In 1010 | 99.999999999% |
| - | 200 | At least 1 in 1020 | at least 99.999999999999999999% |
