# Supplementary material for: PD-1, PD-L1 and cAMP immunohistochemical expressions are associated with worse oncological outcome in patients with bladder cancer
Source: J Cancer Res Clin Oncol. 2022 Aug 16;149(7):3681–90. doi: 10.1007/s00432-022-04262-0 (PMC10314864; doi:10.1007/s00432-022-04262-0)
Supplement: Supplementary file 9 — Supplementary file9 (DOCX 23 KB) [file 432_2022_4262_MOESM9_ESM.docx]

| Suppl. Table 4. Non-synonymous sequenced mutations and corresponding genomic and population information. | | | | | | | | |
| --- | --- | --- | --- | --- | --- | --- | --- | --- |
| Sample | Protein Change | Gene | Nucleotide | Codon Change | Zygosity | Kind of Mutation | dbSNP_ID | MAF |
| Patient 1 | K1491R | ALK | NM_004304.5 | c.4472A>G | Heterozygous | Germline | rs1881420 | 0.30 |
|  | T358I | PIK3CA | NM_000222.3 | c.1073C>T | Heterozygous | Somatic | - | - |
|  | g.423211T>A | ESR1 | NG_008493.2 | g.423211T>A | Heterozygous | Somatic | rs7766585 | 0.25 |
|  | P72R | TP53 | NM_001276760.2 | c.98C>G | Heterozygous | Germline | rs1042522 | 0.45 |
|  | P1170A | ERBB2 | NM_001289937.1 | c.3508C>G | Heterozygous | Germline | rs1058808 | 0.39 |
| Patient 2 | g.423211T>A | ESR1 | NG_008493.2 | g.423211T>A | Heterozygous | Germline | rs7766585 | 0.25 |
|  | KRAS: 3 Prime UTR Variant | KRAS | NC_000012.11 | g.25360224A>C | Heterozygous | Germline | rs61764370 | 0.04 |
|  | P72L | TP53 | NM_001276760.2 | c.98C>T | Heterozygous | Germline | rs1042522 | 0.38 |
|  | E545K | PIK3CA | NM_006218.4 | c.1633G>A | Heterozygous | Somatic | rs104886003 | 0.00 |
| Patient 3 | P832S | KIT | NM_000222.3 | c.2482C>T | Heterozygous | Somatic | - | - |
|  | Q1027* | ERBB2 | NM_001289937.1 | c.2989C>T | Heterozygous | Somatic | - | - |
|  | R521K | EGFR | NM_005228.5 | c.1562G>A | Heterozygous | Somatic | rs2227983 | 0.30 |
|  | P72R | TP53 | NM_001276760.2 | c.98C>G | Heterozygous | Germline | rs1042522 | 0.45 |
|  | P1170A | ERBB2 | NM_001289937.1 | c.3508C>G | Heterozygous | Germline | rs1058808 | 0.39 |
| Patient 4 | M745I | PIK3CA |  | c.2235G>A | Heterozygous | Germline | - | - |
|  | g.423211T>A | ESR1 | NM_000125.4 | c.1369+13777T>A | Heterozygous | Germline | rs7766585 | 0.25 |
|  | R521K | EGFR | NM_005228.5 | c.1562G>A | Heterozygous | Germline | rs2227983 | 0.30 |
|  | T402I | ERBB2 | NM_001289937.1 | c.1205C>T | Heterozygous | Somatic | - | - |
|  | S451C | ERBB2 | NM_001289937.1 | c.3355A>T | Heterozygous | Somatic | - | - |
|  | R1161Q | ERBB2 | NM_001289937.1 | c.*61G>A | Heterozygous | Germline | rs150680317 | 0.00 |
|  | E1195K | ERBB2 | NM_001289937.1 | c.3493G>A | Heterozygous | Somatic | - | - |
|  | K1491R | ALK | NM_004304.5 | c.4472A>G* | Heterozygous | Somatic | rs1881420 | 0.30 |
|  | I391M | PIK3CA | NM_006218.4 | c.1173A>G | Heterozygous | Germline | rs2230461 | 0.06 |
|  | M541L | KIT | NM_000222.3 | c.1621A>C | Heterozygous | Germline | rs3822214 | 0.09 |
|  | EGFR Intron Variant | EGFR | NM_005228.5 | c.2184+19G>A | Heterozygous | Germline | rs17337107 | 0.04 |
|  | KRAS: 3 Prime UTR Variant | KRAS | NC_000012.11 | g.25360224A>C | Heterozygous | Germline | rs61764370 | 0.04 |
|  | S119C | ERBB2 | NM_001289937.1 | c.3355A>T | Heterozygous | Germline | rs773123 | 0.08 |
|  | P72R | TP53 | NM_001276760.2 | c.98C>G | Heterozygous | Germline | rs1042522 | 0.03 |
|  | P1170A | ERBB2 | NM_001289937.1 | c.3508C>G | Heterozygous | Germline | rs1058808 | 0.40 |
| Patient 5 | K1491R | ALK | NM_004304.5 | c.4472A>G* | Heterozygous | Germline | rs1881420 | 0.30 |
|  | R521K | EGFR | NM_005228.5 | c.1562G>A | Heterozygous | Germline | rs2227983 | 0.20 |
|  | P72R | TP53 | NM_001276760.2 | c.98C>G | Heterozygous | Germline | rs1042522 | 0.30 |
|  | P1170A | ERBB2 | NM_001289937.1 | c.3508C>G | Heterozygous | Germline | rs1058808 | 0.40 |
| Patient 6 | K1491R | ALK | NM_004304.5 | c.4472A>G* | Heterozygous | Germline | rs1881420 | 0.30 |
|  | P72R | TP53 | NM_001276760.2 | c.98C>G | Heterozygous | Germline | rs1042522 | 0.30 |
|  | I654V | ERBB2 | NM_001289937.1 | c.1960A>G | Heterozygous | Germline | rs1801201 | 0.01 |
|  | I655V | ERBB2 | NM_001289937.1 | c.1963A>G | Heterozygous | Germline | rs1136201 | 0.19 |
|  | P1170A | ERBB2 | NM_001289937.1 | c.3508C>G | Heterozygous | Germline | rs1058808 | 0.40 |
| Legend: | | | | | | | | |
