# Supplementary material for: PD-1, PD-L1 and cAMP immunohistochemical expressions are associated with worse oncological outcome in patients with bladder cancer
Source: J Cancer Res Clin Oncol. 2022 Aug 16;149(7):3681–90. doi: 10.1007/s00432-022-04262-0 (PMC10314864; doi:10.1007/s00432-022-04262-0)
Supplement: Supplementary file 10 — Supplementary file10 (DOCX 16 KB) [file 432_2022_4262_MOESM10_ESM.docx]

| ***Suppl. Table 5. dbSNP_ID found in data analysis*** | | | | | | | | |
| --- | --- | --- | --- | --- | --- | --- | --- | --- |
| dbSNP_ID |  | ***Pz1*** | ***Pz2*** | ***Pz3*** | ***Pz4*** | ***Pz5*** | ***Pz6*** | ***Frequency*** |
| *rs2227983* | EGFR | 0 | 0 | 1 | 1 | 1 | 0 | 50.0 |
| *rs17337107* |  | 0 | 0 | 0 | 1 | 0 | 0 | 16.7 |
| *rs1801201* | ERBB2 | 0 | 0 | 0 | 0 | 0 | 1 | 16.7 |
| *rs1058808* |  | 1 | 0 | 1 | 1 | 1 | 1 | 83.3 |
| *rs773123* |  | 0 | 0 | 0 | 1 | 0 | 0 | 16.7 |
| *rs150680317* |  | 0 | 0 | 0 | 1 | 0 | 0 | 16.7 |
| *rs1136201* |  | 0 | 0 | 0 | 0 | 0 | 1 | 16.7 |
| *rs7766585* | ESR1 | 1 | 1 | 0 | 1 | 0 | 0 | 50.0 |
| *rs61764370* | KRAS | 0 | 1 | 0 | 1 | 0 | 0 | 33.3 |
| *rs1881420* | ALK | 1 | 0 | 0 | 1 | 1 | 0 | 50.0 |
| *rs3822214* | KIT | 0 | 0 | 0 | 1 | 0 | 0 | 16.7 |
| *rs1042522* | TP53 | 1 | 1 | 0 | 1 | 1 | 1 | 83.3 |
| *rs2230461* | PIK3CA | 0 | 0 | 0 | 1 | 0 | 0 | 16.7 |
| *rs104886003* |  | 0 | 1 | 0 | 0 | 0 | 0 | 16.7 |
| ***Total of rs*** |  | 4 | 4 | 2 | 11 | 4 | 4 |  |
| Legend: | | | | | | | | |
